# Joint Nordic prospective study on human herpesvirus 8 and multiple myeloma risk

**DOI:** 10.1038/sj.bjc.6602751

**Published:** 2005-08-30

**Authors:** R Tedeschi, T Luostarinen, P De Paoli, R E Gislefoss, L Tenkanen, J Virtamo, P Koskela, G Hallmans, M Lehtinen, J Dillner

**Affiliations:** 1 Department of Microbiology-Immunology and Virology, Centro di Riferimento Oncologico, IRCCS, I-33081 Aviano, Italy; 2 Finnish Cancer Registry, Institute for Statistical Epidemiological Cancer Research, FIN-00171 Helsinki, Finland; 3 Institute of Clinical Biochemistry, Rikshospitalet, 0027 Oslo, Norway; 4 Helsinki Heart Study, National Public Health Institute, Helsinki, Finland; 5 Department of Epidemiology and Health Promotion, National Public Health Institute, Helsinki, Finland; 6 Department of Viral Diseases and Immunology, National Public Health Institute, Oulu, Finland; 7 Northern Sweden Health and Disease Study, The Medical Biobank, Umeå University, Sweden; 8 Department of Medical Microbiology, Lund University, University Hospital MAS, Entrance 78, S-20502 Malmö, Sweden

**Keywords:** immunofluorescence, biobank, multiple myeloma, HHV8, nested case–control study

## Abstract

An association between human herpesvirus 8 (HHV8) and multiple myeloma (MM) has been reported, though most studies have not confirmed such association. To follow-up on a previous prospective seroepidemiological study, where HHV8 tended to associate with MM risk, we linked five large serum banks in the Nordic countries with the Nordic cancer registries and 329 prospectively occurring cases of MM were identified, together with 1631 control subjects matched by age and gender. The HHV8 seroprevalences among cases and controls were similar (12 and 15%, respectively) and HHV8 seropositivity did not associate with the risk of MM, neither when considering positivity for lytic antibodies (relative risk (RR)=0.8, 95% confidence interval (CI)=0.5–1.1) nor for latent antibodies (RR=0.6, 95% CI=0.1–2.7). Similar risks were seen when analysis was restricted to case–control sets with at least 2 years lag before diagnosis (RR=0.8, 95% CI=0.5–1.2 and RR=0.9, 95% CI=0.1–4.2). In conclusion, the data indicate that HHV8 infection is not associated with MM.

Rettig *et al* (1997) reported that HHV8 DNA was present in bone marrow dendritic cells of multiple myeloma (MM) patients, but subsequent studies had inconsistent findings (Olsen *et al*, 1998; Beksac *et al*, 2001; Patel *et al*, 2001; Hermouet *et al*, 2003). Several studies found that HHV8 antibodies did not associate with MM (MacKenzie *et al*, 1997; Masood *et al*, 1997; Parravicini *et al*, 1997; Bouscary *et al*, 1998; Perna *et al*, 1998; Santarelli *et al*, 1998; Ablashi *et al*, 2000), although one study found an association (Gao *et al*, 1998). However, the large amounts of myeloma proteins in MM patients' sera may induce systematic measurement biases in serological assays. We previously studied MM in a prospective study nested in a cohort of 39 057 healthy Finnish subjects (Tedeschi *et al*, 2001a). Seropositivity for lytic and latent HHV8 antigens was found in 16 and 0.4% of the population, and had a nonsignificant tendency to associate with MM risk (relative risk (RR)=2.02, 95% confidence interval (CI)=0.94–4.33 and RR=10.00, 95% CI=0.91–110.29, respectively), but the statistical power of our study was limited. We have therefore performed a new study with the same study design and HHV8 analyses, but on an independent and much larger material.

## MATERIALS AND METHODS

### Cohorts

The study base was constituted of five prospectively followed cohorts covering about 1 133 000 individuals who had donated serum and/or plasma samples.

*The Finnish Maternity Cohort* contains serum samples from 700 000 pregnant women attending maternity clinics (about 98% of all those pregnant since 1983) in Finland for serological screening of rubella immunity, HIV and hepatitis in week 14 of pregnancy (Kibur *et al*, 2000).

*The Helsinki Heart Study* tested whether treatment with gemfibrozil (a fibric acid derivative) reduces the incidence of coronary heart disease (CHD) in middle-aged, dyslipidaemic men, the volunteers for which were selected from men aged 40–55 years, employed by two government agencies and five industrial companies, and living in different parts of Finland. About 19 000 men were enrolled, during 1980–1982.

*The Alpha-Tocopherol, Beta-Carotene Cancer Prevention* (*ATBC*) *Study* evaluated whether daily supplementation with alpha-tocopherol or beta-carotene would reduce the incidence of lung cancer and other cancers (ATBC Cancer Prevention Study Group, 1994).

*The Janus project* was started in 1973 to collect and store blood samples from healthy persons for later scientific use. Serum samples from population-based invitational health examinations from the whole Norway and from the Red Cross blood donors in capital Oslo and surrounding areas are included in the biobank, which now covers about 315 000 persons, 10% of whom are blood donors who give 20 ml of extra blood every other year to the Janus project. At the first donation a declaration of consent is signed, allowing use of the sera for anonymous cancer research.

*The Northern Sweden Health and Disease Study* includes three major subcohorts, including the subcohort from an intervention project that invites all individuals resident in the Västerbotten county of Northern Sweden aged 40, 50 and 60 (during 1985–1995, also those aged 30) for a health-promoting visit and to donate a blood sample (fractionated into plasma, erythrocytes and buffy-coat, all stored at −80°C) for later research purposes. The project started in 1985 and currently includes about 70 000 individuals.

### Identification of cases and controls

A total of 329 prospectively occurring cases of MM were identified in the Nordic joint study base, all diagnosed more than a month after the serum sampling. Five control subjects that had not developed MM during and with equal length of follow-up were matched to each case for biobank, sex, age at serum sampling (±2 years), time of withdrawal of serum sample (±2 months) – and in Norway also for county of residence – and were selected at random among all eligible controls. If controls could not be found, the matching criteria were widened in successive steps of ±1 year of age and ±1 month of storage until controls were found. The maximum age difference between a case and any matching controls at the serum sampling was 2.1 years and the mean age difference was 0.9 years (standard deviation 0.6 years). The date of serum sampling differed on average by 0.9 months between a case and its controls (standard deviation 0.8 months). The samples were analysed in batches, each containing max 54 samples (typically containing nine cases and their 45 matched controls), with the sample order assigned randomly. The record of randomisation contained an 8–9-digit number consisting of biobank code (one digit), running number (3–4 digits), batch number (two digits), and location within the batch (two digits).

All serum samples were sent frozen for laboratory analysis at the Centro di Riferimento Oncologico in Aviano, Italy, where they were analysed by immunofluorescence for the presence of HHV8 antibodies by experienced laboratory personnel. The analysing laboratory was blinded to all and any information about the sample. In all, 120 serum samples from the Helsinki Heart Study, 185 serum samples from the Finnish Maternity Cohort, 258 plasma samples from the Västerbotten study, 179 serum samples from the ATBC Study from Finland and 1218 serum samples from the Janus project in Norway were assessed. Samples from 11 cases and 52 controls in the Helsinki Heart Study had accidentally been subjected to an additional freeze–thawing cycle.

### Laboratory methods

HHV8 antibodies were determined using mouse monoclonal antibody enhanced immunofluorescence assay (mIFA) (Lennette *et al*, 1996) using the BCBL-1 cell line, as described elsewhere (Tedeschi *et al*, 2001a). Specimens were screened at a 1/10 dilution and positive samples were titrated up to 1/640. The samples were always analysed in batches of up to 54 samples derived from the same biobank. Each batch was tested together with positive (from HHV8-positive Italian KS patients) and negative (healthy HHV-8 negative donors) serum controls. All samples were also tested with a negative control cell line (Ramos, Burkitt lymphoma cells) and sera reacting with Ramos cells were scored as not analysable.

In all, 376 serum samples (94 MM cases and 282 controls) were also assessed for HHV8 DNA using a sensitive real-time (TaqMan) PCR assay (Heid *et al*, 1996) that amplifies a 67 bp amplicon in the HHV8 minor capsid protein gene (open reading frame 26). DNA was extracted using the QiAmp Blood Kit (Qiagen Gmbh, Hilden, Germany) from 50 *μ*l serum into 50 *μ*l distilled water. The number of genome equivalents in each sample was calculated from an EBV DNA standard (Enbom *et al*, 2001). The standard curve was created using the ABI PRISM 7900 Sequence Detection System software (2.1.1v, Applied Biosystems, Foster City, CA, USA), by plotting the *C*_t_ values against each known concentration of the EBV standard. Reaction conditions were as reported previously (Tedeschi *et al*, 2001b). Negative control water samples (for EBV and for HHV8), positive control HHV8 DNA extracted from culture supernatant of the BCBL1 cells and negative control from the serum sample of an HHV8 seronegative healthy donor were included in triplicate on each plate. The samples were analysed in triplicates, including one inhibition control to which 0.5 *μ*l of the positive control was added to control for inhibitory substances in the samples.

### Statistical analysis

The laboratory results were transmitted to the Finnish Cancer Registry, where the code was broken and the RR of developing MM given HHV8 seropositivity was estimated by exact conditional logistic regression with LogXact 5 software (Cytel Software Corporation, Cambridge, MA, USA). The RRs were adjusted for the accidental extra freeze–thawing. The association between latent and lytic seroreactivity was measured by the Spearman rank-order correlation coefficient.

## RESULTS

### Detection of IgG antibodies to HHV8

Altogether, 277 out of 1960 (14%) and 18 out of 1960 (1%) serum samples were HHV8 positive in IFA for lytic antibodies and for the latent antibodies, respectively, with serum titres ranging from 1 : 10 to ⩾1 : 640. The correlation between lytic and latent seroreactivity was 0.22 (*P*=0.004) among cases and 0.19 (*P*<0.001) among controls, respectively. There was no difference in HHV8 seroreactivity, considering males or females (Table 1). Among the different cohorts studied, we found a significantly higher HHV8 seroprevalence (IFA lytic was 27.9% and IFA latent was 1.7%, respectively) among the samples from the ATBC Study (a Finnish cohort consisting only of men, all heavy smokers).

In all, 94 serum samples from MM cases and 282 from the controls, randomly selected, were also assessed for HHV8 DNA using a quantitative real-time PCR assay. Viral DNA was undetectable in all these samples tested and the other samples in the study were therefore not tested for HHV8 DNA.

The RRs of developing MM in case of HHV8 seropositivity were 0.8 (95% CI=0.5–1.1) and 0.6 (95% CI=1.0–2.7), respectively, for the presence of lytic and latent HHV-8 antibodies (Table 1); no differences were observed when the different serum banks were evaluated separately (Table 1).

To investigate whether the presence of as yet undiagnosed MM may have affected the results, we also analysed the data with the samples taken less than 2 years before diagnosis of MM was excluded, as well as by dichotimising the study into case–control sets with shorter or longer lag between enrolment and diagnosis. There were no noteworthy differences in results depending on lag (Table 2).

## DISCUSSION

The prospective biobank-based study design, when used in countries with complete nationwide case ascertainment, minimises most of the epidemiological sources of bias. For example, reverse causality biases are not likely to occur with the long follow-up times between blood draw and diagnosis of cancer, and selection biases due to incomplete attendance rate or inadequate study base definition are also unlikely.

We evaluated whether HHV8 infection was associated with an increased risk for MM, using the same prospective biobank-based study design and analyses as in our previous study that had found a nonsignificant tendency of association. We found no evidence of increased risk of MM associated with HHV8 seropositivity. As our current study is by far the largest ever prospective study on MM, the data suggest that HHV-8 is not associated with risk of MM. This conclusion is in line with several serological studies largely performed on different MM populations (Cottoni and Uccini, 1997; Mackenzie *et al*, 1997; Marcelin *et al*, 1997; Masood *et al*, 1997; Whitby *et al*, 1997; Olsen *et al*, 1998). The single study that found HHV8 antibodies to be common among MM patients (Gao *et al*, 1998) used a sensitive immunoblotting method for detection of antibodies against the recombinant minor capsid antigen and latent nuclear antigen of HHV8. The present study did not use immunoblotting for measuring HHV8 antibodies, because our previous prospective study of MM did include immunoblotting against the viral antigens ORF65, ORF73 and ORFK8.1A, but found no association (Tedeschi *et al*, 2001a). The possibility that lack of association of the serological response to HHV8 and MM risk patients may reflect the relatively immunodeficient state typically associated with this malignancy is not likely, as the samples were taken up to 25 years before diagnosis of MM. The lack of association could conceivably be explained if MM is associated with a specific immune defect that prevents the generation of serologic responses to HHV8 or if it is associated with a unique strain of HHV8 that has a different immunogenic profile compared to the HHV8 strains found in KS (Berenson *et al*, 2000). However, in a recent study (Zhu *et al*, 2002), DNA sequences of three antigenic regions (ORF65, ORF73 and ORFK8.1) and the transforming hypervariable K1 ORF of HHV8 were analysed in fresh bone marrow cells, bone-marrow-derived dendritic cells and bone marrow stromal cells, and were shown to be present in MM patients and normal individuals at similar frequencies.

In summary, the tendency for association between HHV-8 and MM in our previous smaller study could not be confirmed and our results provide evidence against an involvement of HHV8 in MM development.

## Figures and Tables

**Table 1:** RR of developing MM among subjects seropositive for HHV-8

	**MM on follow-up**	**Healthy on follow-up**	**RR**	**95% CI**
IFA lytic				
No	290	1393	1	
Yes	39	238	0.8	(0.5–1.1)
				
IFA latent				
No	327	1615	1	
Yes	2	16	0.6	(0.1–2.7)
				
*Females*				
IFA lytic				
No	106	544	1	
Yes	18	75	1.2	(0.7–2.2)
				
IFA latent				
No	123	611	1	
Yes	1	8	0.6	(0.0–4.7)
				
*Males*				
IFA lytic				
No	84	849	1	
Yes	21	163	0.6	(0.3–1.0)
				
IFA latent				
No	204	1004	1	
Yes	1	8	0.6	(0.0–4.7)
				
*FMC*				
IFA lytic				
No	29	131	1	
Yes	2	23	0.4	(0.0–1.7)
				
IFA latent				
No	31	152	1	
Yes	0	2	0^a^	(0–27)
				
*HHS*				
IFA lytic				
No	16	80	1	
Yes	4	20	1.0	(0.2–3.7)
				
IFA latent				
No	20	99	1	
Yes	0	1	0^b^	(0–190)
				
*ATBC*				
IFA lytic				
No	25	104	1	
Yes	7	43	0.7	(0.2–1.9)
				
IFA latent				
No	31	145	1	
Yes	1	2	2.5	(0.0–4.8)
				
*JANUS*				
IFA lytic				
No	183	904	1	
Yes	20	111	0.9	(0.5–1.5)
				
IFA latent				
No	202	1006	1	
Yes	1	9	0.6	(0.0–4.0)
				
*Umeå*				
IFA lytic				
No	37	174	1	
Yes	6	41	0.7	(0.2–1.8)
				
IFA latent				
No	43	213	1	
Yes	0	2	0^a^	(0–27)

RR=relative risk; MM=multiple myeloma; CI=confidence interval; IFA=immunofluorescence assay; FMC=Finnish Maternity Cohort; HHS=Helsinki Heart Study; Umeå=The Northern Sweden Health and Disease Study; ATBC=Alpha Tocopherol Beta Carotene prevention study; Janus=The Janus biobank; Method: exact conditional logistic regression, adjustment for accidental freeze–thawing.

a Median unbiased estimate=2.1.

b Median unbiased estimate=5.0.

**Table 2:** RR of developing multiple myeloma by lag between sampling and diagnosis

	**MM on follow-up**	**Healthy on follow-up**	**RR**	**95% CI**
*Cases with lag >24 months:*				
IFA lytic				
No	267	1288	1	
Yes	37	221	0.8	(0.5–1.2)
				
IFA latent				
No	302	1498	1	
Yes	2	11	0.9	(0.1–4.2)
				
*Cases with <median lag (121 months)*				
IFA lytic				
No	144	668	1	
Yes	20	143	0.6	(0.4–1.1)
				
IFA latent				
No	163	798	1	
Yes	1	13	0.4	(0.0–2.6)
				
*Cases with at least median lag (121 months)*				
IFA lytic				
No	146	725	1	
Yes	19	95	1.0	(0.5–1.7)
				
IFA latent				
No	164	817	1	
Yes	1	3	1.7	(0.0–2.1)

RR=relative risk; MM=multiple myeloma; CI=confidence interval; IFA=immunofluorescence assay.

Method: exact conditional logistic regression, adjustment for freeze–thawing.
